# Genetic and Immunological Insights into Tick-Bite Hypersensitivity and Alpha-Gal Syndrome: A Case Study Approach

**DOI:** 10.3390/ijms26020680

**Published:** 2025-01-15

**Authors:** Pavle Banović, Dejan Jakimovski, Dragana Mijatović, Ivana Bogdan, Verica Simin, Jasmina Grujić, Svetlana Vojvodić, Nada Vučković, Kinga Lis, Eleftherios Meletis, Polychronis Kostoulas, Marija Cvetkova Mladenovska, Angélique Foucault-Simonin, Sara Moutailler, Lourdes Mateos-Hernández, Alejandro Cabezas-Cruz

**Affiliations:** 1 Clinic for Lyme Borreliosis and Other Tick-Borne Diseases, Department of Prevention of Rabies and Other Infectious Diseases, Pasteur Institute Novi Sad, 21000 Novi Sad, Serbia; 2 Department of Microbiology with Parasitology and Immunology, Faculty of Medicine in Novi Sad, University of Novi Sad, 21000 Novi Sad, Serbia; 3 Diagnostics and Laboratory Research Task Force, Balkan Association for Vector-Borne Diseases, 21000 Novi Sad, Serbia; draganav77@gmail.com (D.M.); ivana.basaric@gmail.com (I.B.); vericasimin071@gmail.com (V.S.); jasmina.grujic@mf.uns.ac.rs (J.G.); 4 Faculty of Medicine, Ss. Cyril and Methodius University in Skopje, 1000 Skopje, North Macedonia; dejan.jakimovski@medf.ukim.edu.mk; 5 University Clinic for Infectious Diseases and Febrile Conditions, 1000 Skopje, North Macedonia; 6 Clinical medicine Task Force, Balkan Association for Vector-Borne Diseases, 21000 Novi Sad, Serbia; 7 Department for Research & Monitoring of Rabies & Other Zoonoses, Pasteur Institute Novi Sad, 21000 Novi Sad, Serbia; 8 Department of Microbiology, Pasteur Institute Novi Sad, 21000 Novi Sad, Serbia; 9 Department of Transfusiology, Faculty of Medicine in Novi Sad, University of Novi Sad, 21000 Novi Sad, Serbia; svetlana.vojvodic@mf.uns.ac.rs; 10 Blood Transfusion Institute of Vojvodina, 21000 Novi Sad, Serbia; 11 Pathology and Histology Centre, Clinical Centre of Vojvodina, Novi Sad, Department of Pathology, Faculty of Medicine in Novi Sad, University of Novi Sad, 21000 Novi Sad, Serbia; nadavuckovic@yahoo.com; 12 Department of Allergology, Clinical Immunology and Internal Diseases, Collegium Medicum in Bydgoszcz, Nicolaus Copernicus University in Toruń, Ujejskiego 75, 85-168 Bydgoszcz, Poland; kinga.lis@cm.umk.pl; 13 Faculty of Public and One Health, University of Thessaly, 43100 Karditsa, Greece; emeletis@outlook.com (E.M.); pkost@uth.gr (P.K.); 14 Epidemiology and Biostatistics Research Task Force, Balkan Association for Vector-Borne Diseases, 21000 Novi Sad, Serbia; 15 Orthopedic Surgery, Anesthesiology and Intensive Care and Emergency Center, University Clinic for Traumatology, 1000 Skopje, North Macedonia; cvetkovamarijam90@gmail.com; 16 Agence Nationale de Sécurité Sanitaire de l’alimentation, de l’environnement et du Travail, l’Institut National de Recherche Pour l’agriculture, l’alimentation et l’environnement, Ecole Nationale Vétérinaire d’Alfort, UMR Biologie Moléculaire et Immunologie Parasitaires, Laboratoire de Santé Animale, F-94700 Maisons-Alfort, France; angelique.foucaultsimonin@anses.fr (A.F.-S.); sara.moutailler@anses.fr (S.M.); lourdes.mateos@vet-alfort.fr (L.M.-H.)

**Keywords:** alpha-gal syndrome, genetic predisposition, ticks, HLA polymorphisms, tick-bite hypersensitivity, fixed drug reaction

## Abstract

Tick-bite hypersensitivity encompasses a range of clinical manifestations, from localized allergic reactions to systemic conditions like alpha-gal syndrome (AGS), an IgE-mediated allergy to galactose-α-1,3-galactose (α-Gal). This study investigated the clinical, molecular, immunological, and genetic features of two hypersensitivity cases. Two cases were analyzed: a 30-year-old woman with fixed drug reaction (FDR)-like hypersensitivity and a 10-year-old girl with AGS exhibiting borderline α-Gal-specific IgE. Diagnostic methods included allergen-specific IgE quantification, HLA genotyping, histopathological examination, and the molecular detection of tick-borne pathogens using microfluidic PCR. Case I demonstrated histopathological features of chronic lymphocytic inflammation and eosinophilic infiltrates, with HLA-B13 and DRB113 alleles indicating genetic susceptibility to hypersensitivity, while histological findings suggested a localized FDR-like reaction. Case II exhibited borderline α-Gal-specific IgE, resolving completely with a mammalian-free diet. The presence of HLA-DRB101 and DQB1*05 in the second patient indicated a genetic predisposition to AGS and other atopic conditions. No infectious etiology was identified in either case. These findings emphasize the heterogeneity of tick-related hypersensitivity and the importance of HLA genotypes in susceptibility. Comprehensive molecular, immunological, and genetic profiling offers valuable insights into the mechanisms of hypersensitivity, supporting personalized approaches for the diagnosis and management of tick-induced allergic conditions.

## 1. Introduction

Alpha-gal syndrome (AGS) is tick-bite-associated allergy to mammalian meat, caused by IgE acting against galactose-alpha-1,3-galactose (aGal) [1]. The syndrome can manifest in the form of delayed or acute anaphylaxis after exposure to aGal epitopes on mammalian meat, tick saliva, and specific drugs (e.g., cetuximab) [2]. In the case of mammalian meat consumption, AGS differs from majority of IgE-mediated food allergies (e.g., peanut, tree nuts, and seafood allergies), as manifestations do not occur rapidly after allergen consumption [2,3]. Classic AGS manifestation comprises of skin reactions (chives/angioedema) occurring 2–6 h after the ingestion of mammalian meat with the detection of anti-α-Gal IgE (≥0.1 IU/mL or <2% of the total IgE), where clinical improvement is observed after the implementation of a diet without mammalian-origin nutrients [4]. AGS is reported worldwide and is most commonly associated with prior exposure to tick bites. Additionally, ABO blood type, sex, alcohol consumption, exposure to cats, and helminth infection have been proposed as contributing co-factors that may influence AGS manifestation [2]. On the other hand, persons suffering from AGS are identified as more prone to developing atherosclerosis and consequential cardiovascular complications [5].

The major histocompatibility complex (MHC) in humans is a genetic region that includes genes that encode the synthesis of human leukocyte antigens (HLAs). The HLA complex has a major role in antigen presentation and can activate and amplify the downstream response of humoral and cellular immunity [6]. Numerous studies have been able to determine the link between HLA genotypes and hypersensitivity to specific antigens (e.g., components of food, drugs, insects, animals, etc.) [7]. HLA class II most often plays a crucial role in the initial presentation of antigens to CD4+ T cells and the direction of Th2-B interaction that results in IgE production and secretion [8,9]. Data on HLA polymorphisms in individuals with tick-related hypersensitivity are currently unavailable, highlighting an objective need for foundational research to explore risk factors for tick-related hypersensitivity through a multimodal approach.

In this study, we present a comparative analysis of AGS and local hypersensitivity reactions following tick bites, integrating clinical, microbiological, immunological, and molecular findings in affected individuals.

## 2. Results

### 2.1. Description of Hypersensitivity Cases

#### 2.1.1. Case I

A woman in her thirties, residing in Skopje, North Macedonia, sought treatment at the Infectious Diseases Clinic in February 2023 for a persistent itching rash on her left upper abdomen. This rash had appeared following a tick bite she sustained in Central Greece six months earlier, in September 2022. Upon returning from Greece, the patient discovered a tick attached to her left upper abdomen, which she promptly had removed at the emergency department in Skopje. Subsequently, she noticed the formation of a pruritic papule at the site of the bite. Two months later, in November 2022, the papule gradually developed into a pruritic rash that extended beyond the initial bite area. After consulting her primary care physician, she intermittently applied betamethasone/gentamicin 0.5/1.0 mg topical cream over the next three months (November to February 2023). However, despite this treatment, the rash persisted, displaying periodic changes in color and intensity of itching, seemingly unaffected by the topical medication.

**Figure 1:**
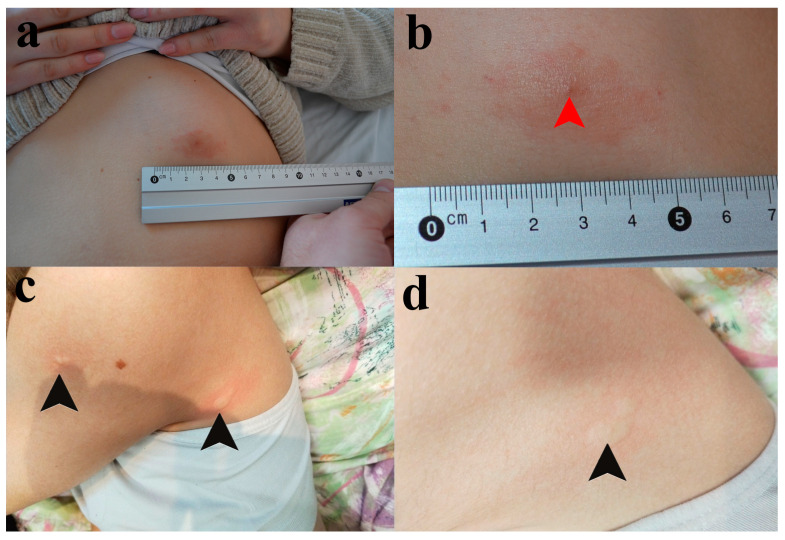
Skin lesions presented in Patients I and II. (**a**) The pruritic red patch on the skin of the frontal abdominal wall in Patient I. (**b**) The tick infestation site (red arrowhead). (**c**) The skin lesions of Patient II. Black arrowheads are pointed at wheals on the back side of the axilla and proximal portion of the upper arm. (**d**) An individual wheal on the back side of the axilla.

Upon examination at our clinic on 14 February 2023, the patient continued to use betamethasone/gentamicin 0.5/1.0 mg topical cream. We observed an oval, pruritic, and erythematous plaque with mild infiltration, slightly raised edges, and a central point of entry, measuring approximately 4 cm in diameter, located in the left upper abdominal quadrant (See Figure 1a,b).

The patient’s medical history revealed a pollen allergy, and her family’s history included hypersensitivity to mosquito bites [10], and recurrent uninvestigated allergic respiratory and dermatological reactions. Additionally, she reported experiencing frequent digestive issues, typically manifesting as stomach aches occurring 30 min to 2 h after consuming red meat.

Blood samples were collected for biochemical analysis, complete blood count, ABO blood typing (see Section 4.1.1 and Section 4.2.2), and assessment of seroreactivity to *Borrelia burgdorferi* sensu lato complex and spotted fever group *Rickettsia* (SFGR) (see Section 4.2.3). Suspecting an allergy to α-Gal, a blood sample was obtained for the quantification of IgE against α-Gal, and seven other meat-specific allergens (see Section 4.2.1), as well as for HLA genotyping (see Section 4.4.1).

Furthermore, a skin biopsy was conducted at the lesion site (both at the center and periphery) for histopathological examination (see Section 4.5) and the detection of DNA from 27 tick-related bacterial species and eight tick-related parasite groups using the microfluidic PCR technique (see Section 4.3).

A treatment regimen consisting of doxycycline 100 mg twice daily and desloratadine 5 mg daily for 14 days was initiated. The patient was instructed to adhere to a strict dietary regimen avoiding meat and animal protein-containing products.

Two weeks after the introduction of a restrictive dietary regimen and treatment with doxycycline/desloratadine, no changes in lesion size and shape were observed. The patient was advised to continue with a restrictive dietary regimen. Doxycycline/desloratadine treatment was discontinued and the local application of clobetasol propionate 0.05% was introduced. Four weeks later, the lesion size decreased by 50% and the patient reported that the itching sensation was less frequent. Nevertheless, the lesion relapsed shortly after the application of clobetasol propionate was discontinued. Finally, pimecrolimus cream 1% was introduced, reducing the lesion size by approximately 90% after six weeks.

#### 2.1.2. Case II

A 10-year-old girl was admitted to Pasteur Institute Novi Sad due to the continuous emergence of hives across the body at the sites of previous tick infestations. The itching sensation was constant during the day and more intense during the night. Their parents noticed that these lesions usually develop after the child is exposed to mosquito or tick bites, and that hives last for several days, after which they gradually resolve. In addition, the patient complained about a stomach ache that has lasted for at least six years. Within that period, the patient was hospitalized on several occasions and examined by a gastroenterologist, a pediatrician, an endocrinologist, and an infectious disease specialists, as well as radiologists, but without a definite etiological diagnosis. Their parents reported that the stomach ache occurs almost every day, leading them to change her diet strictly to food that is locally produced. Nevertheless, they had not observed a reduction in stomach ache-related complaints, and the patient complained that she now generally tries to avoid eating when possible.

During a physical examination, urticaria was noticed in several foci across the body, including the most recent sites of mosquito bites, favoring the diagnosis of recall urticaria (Figure 1c,d). Although the girl was not showing signs of malnutrition, her skin was pale as she was avoiding going outside in order to prevent another tick or mosquito bite.

Routine biochemical analyses, ABO typing, and complete blood count were ordered (see Section 4.1.1 and Section 4.2.2), including the assessment of seroreactivity to the *Borrelia burgdorferi* sensu lato complex and spotted fever group *Rickettsia* (SFGR) (see Section 4.2.3). Another blood sample was collected for the blood type determination and detection of DNA from 27 tick-related bacterial species and eight tick-related parasite groups using the microfluidic PCR technique (see Section 4.3). Since recall urticaria was previously described as potential sign of AGS [11], a blood sample was obtained for the quantification of total IgE and IgE against α-Gal, and seven other meat-specific allergens (see Section 4.2.1), as well as for HLA genotyping (see Section 4.4.1).

The patient and their parents were instructed to adhere to a strict dietary regimen—food of animal origin can only be from poultry or fish, with no restrictions related to plant-based food. During a control exam, the patient reported that the pain in their stomach resolved completely 3–4 days after the new dietetic regimen was introduced. Physical examination revealed that her skin was not pale as previously noted, she was able to sit straight, and her body mass increased by 2 kg. Although recall urticaria was still developing occasionally, the intensity of the itching sensation was lower and more tolerable.

### 2.2. Routine Laboratory Analyses and ABO Blood Typing

For both patients, results of the laboratory examination including CBC, CRP, AST, ALT, CK, LDH, and serum creatinine values were unremarkable. Patient I and Patient II were affiliated with blood type O.

### 2.3. Serological Analyses

#### 2.3.1. Titers of Allergen-Specific and Total IgE

An analysis of IgE specific to eight examined allergens showed unremarkable results in Patient I, with a total IgE level of 3.99 kU/L (Table 1). On the other hand, anti-α-Gal IgE was found in Patient II (0.20 kUA/L), contributing to 2% of the total IgE present in the serum sample (Table 1).

#### 2.3.2. Examination of Exposure to *Rickettsia* spp. and *Borrelia* spp.

No seroreactivity was detected against *Borrelia* spp. and *Rickettsia* spp. in Patient I. Although Patient II was also non-reactive to *Rickettsia* spp. in IgM and IgG, Borrelia-reactive IgG was detected via ELISA. A second tier test showed strong reactivity against bands containing p41 antigen, as well as weak reactivity to OspC from B. garinii and B. spielmanii, suggesting previous exposure to these members of the Borrelia burgdorferi sensu lato complex. Strong reactivity to the p41 band in an immunoblot analysis suggested that a positive IgG anti-Borrelia finding detected via ELISA may be the consequence of the cross-reaction, where antibodies generated against flagellar structures of other bacteria (e.g., *Helicobacter pylori*, *Escherichia coli*, etc.) bind to the flagellar protein of Borrelia.

### 2.4. Molecular Detection of Tick-Borne Pathogens in Skin Lesion and Complete Blood Samples

Microfluidic PCR analysis of 27 bacterial and eight parasitic tick-borne microorganisms showed no positive findings in the skin biopsy sample from Patient I and the complete blood samples of both patients.

### 2.5. HLA Class I and II Genotyping

Both patients had at least one risk HLA-B, HLA-DRB1, or HLA-DQB1 allele connected to a hypersensitivity reaction or susceptibility to autoimmune/auto-inflammatory diseases. HLA class I and II genotypes of patients with hypersensitivity reactions after tick bites are presented in Table 2.

### 2.6. Histological Findings in Skin Biopsy of Patient I

**Figure 2:**
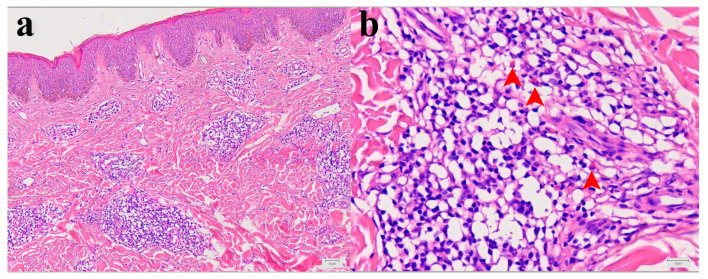
Morphology of Patient I skin lesion biopsy. (**a**) Skin biopsy with mild epidermal hyperkeratosis, dermal edema, and chronic, moderate, perivascular, and inflammatory infiltrate. Capillary blood vessels are slightly dilated (hematoxylin and eosin (H&E), X100). (**b**) Higher magnification of inflammatory infiltrate with few eosinophilic granulocytes (red arrowheads) within dominant lymphocytic infiltrate (H&E, X400).

Histopathological analysis showed mild epidermal hyperkeratosis, dermal edema, and a moderately dense, chronic inflammatory infiltrate, containing some eosinophilic granulocytes, unevenly distributed but mainly perivascular oriented, throughout the thickness of the involved dermis and at the border with subcutaneous fatty tissue. The capillary blood vessels were dilated. The findings corresponded to chronic superficial and deep perivascular lymphocytic infiltrate with eosinophils and minimal or no epidermal alteration. Such rather nonspecific tissue reactions can be seen in a various skin reactions such as in a dermal hypersensitivity reaction/response and in a urticarial hypersensitivity reaction (Figure 2a,b).

## 3. Discussion

Cases of tick-bite-induced hypersensitivity in humans are reported worldwide, but the underlying mechanisms and risk factors for the development of this condition are not completely understood. According to anamnestic data, clinical manifestation, laboratory findings, and the response to treatment/a restrictive diet, the tick-bite-induced hypersensitivity presented in Patient I was finally diagnosed as having an FDR, while Patient II was diagnosed with AGS.

The histopathological changes observed in Patient I were crucial for establishing the final diagnosis, as other laboratory parameters yielded unremarkable results. Changes in the skin biopsy were comparable, or even equivalent, to those observed in clinically manifested papular dermatitis or urticaria, terms often used synonymously with arthropod bite reactions and drug-induced hypersensitivity syndrome [12]. Lesion morphology is suggestive of chronic processes and it is not equivalent to findings in a skin biopsy after tick removal, where eosinophils are the most dominant leukocyte population [13]. On the other hand, similar skin changes were found more than 25 years ago in two lumbermen from France who reported to a clinic due to persistent skin reactions following tick bites [14].

An FDR is considered to be a type IV hypersensitivity reaction and it is mediated by CD8+ T-lymphocytes, which are mostly found in the basal layer of the epidermis affected by the FDR lesion. An FDR may occur at the same location as a previous skin trauma, such as a burn, tick bite, or venipuncture [15]. According to our findings, there is a high possibility that the tick bite in Patient I caused an immune reaction equivalent to the one that occurs after the ingestion of medication and induced skin changes that correspond to an FDR [16].

Several forms of drug hypersensitivity reactions, including FDRs, are associated with the HLAs, as the reaction is triggered when HLAs present modified or altered peptide sequences to T lymphocytes [17]. The patient with an FDR presented here had HLA-A30 B13 Cw6, HLA-B*13, and HLA DRB1*13-DQB1*06 haplotypes, which make her susceptible to an FDR induced by trimethoprim/sulfamethoxazole and dapsone, as well as to aspirin-induced urticaria, respectively [18,19,20,21]. Considering that our patient has HLA markers that are connected to drug-induced hypersensitivity, there is a possibility that she is genetically predisposed to the appearance of skin changes that resemble an FDR after a tick bite.

In the case of AGS, clinical manifestations are the consequence of a delayed hypersensitivity reaction. The reaction is triggered by IgE binding to α-Gal present in hematophagous arthropods, animal-based nutrients, chimeric monoclonal anti-EGFR IgG1 used for carcinoma treatment (i.e., cetuximab), and pharmaceuticals/medical devices containing animal-based products (e.g., anti-venoms and gelatin-containing vaccines) [2,22].

Although AGS occurs in α-Gal-sensitized individuals, not all persons with anti-α-Gal IgE will develop the syndrome. Accordingly, the titer of anti-α-Gal IgE cannot be used for the prediction of AGS severity in an individual [23]. This fact supports the hypothesis that α-Gal-sensitization in humans may be part of acquired tick resistance (ATR) [8,24] given that humans are not natural hosts for ticks [8]. The disease-escaping ability that is achieved through hypersensitivity toward a specific allergen is defined as allergic klenducity, and it is described in several animal species [8]. Accordingly, AGS can be observed as a dysbalance of a non-specific immune response against ticks and α-Gal-expressing microorganisms [8].

Due to the specific structure of the α-Gal epitope and its similarity to the blood type B antigen, persons affiliated to blood type B are considered to be less prone to AGS given that they do not produce anti-B antibodies, while anti-α-Gal antibodies are produced in a lesser amount [2]. The AGS patient described here has blood type O, making her competent to form a potent humoral response against α-Gal.

The global burden of AGS is unknown since a large proportion of cases are undiagnosed due to non-specific and inconsistent symptoms or a lack of awareness among medical professionals [25]. An additional problem is the existence of atypical AGS, which manifests more commonly in children and is characterized by a shorter delay in symptom onset, low titers of anti-α-Gal IgE, and/or gastrointestinal symptoms without skin involvement [4].

The AGS patient described here (i.e., Patient II) was underdiagnosed for at least six years since symptoms onset. Major contributing factors for the delay in final diagnosis were he absence of typical skin manifestations after the consummation of nutrients containing mammal meat or products, where complaints were only related to abdominal pain and cramps after almost every meal. Isolated gastrointestinal symptoms in AGS patients are not considered to be a common manifestation and their prevalence varies from 20 to 78%, depending on the study group cohort [1]. Suspicion for AGS was raised when the patient presented with recall urticaria after a tick bite, since this condition was linked with susceptibility to AGS [10,26]. Although the titer of anti-α-Gal IgE in the patient was 0.20 kUA/L, the percentage of those specific antibodies in the total IgE population was 2%, making this laboratory finding borderline. This finding carries valuable clinical information and underscores the importance of viewing laboratory findings as part of a spectrum, which facilitates nuanced risk assessment and personalized patient management. More precisely, the dichotomization of the output of a continuous biomarker (i.e., the titer of anti-α-Gal IgE) discards valuable information, since the distance between the output and the cut-off is not taken into consideration during test interpretation. For example, values of 0.2 kUA/L and <0.1 kUA/L carry the same information, even though the first one is closer to the cut-off value. Even though the output will be considered as ‘‘negative’’, the finding also needs to be taken with caution, since it is close to the threshold. The finding of a relatively low anti-α-Gal IgE titer could be explained by the fact that pediatric patients do not achieve high levels of these antibodies when compared to adults [27]. The AGS diagnosis in Patient II is supported by the fact that complete remission was reached (including the resolution of recall urticaria) after the introduction of a restrictive diet, where all mammalian-based food was omitted. In addition, a negative PCR and serological findings favor non-infectious etiology.

The allele DRB1*01 found in the AGS patient is considered to be a risk factor for susceptibility to cow milk and egg allergies [28]. In addition, DQB1*05 and DRB1*01 alleles are strongly and positively associated with IgE sensitization to pollen allergens, which means there is a possibility that our AGS patient has an atopic constitution [9].

The HLA class I typing of the AGS patient showed the presence of the HLA B*27 allele, which is highly correlated with the development of inflammatory rheumatologic conditions, generally known as spondyloarthritis [29]. In addition, the HLA-C phenotype found in the AGS patient showed C*02 and C*12 alleles, suggesting susceptibility to severe psoriasis [30]. We did not find any significant correlation related to the symptoms and nature of hypersensitivity observed in Patient II and the alleles within the HLA-A. Our findings provide preliminary evidence that HLA class II loci could serve as AGS susceptibility genetic markers, warranting further investigation into the possible association of AGS with HLA- B27. Data related to the history of skin changes associated with the intake of certain drugs can be important when establishing a final diagnosis.

## 4. Materials and Methods

### 4.1. Laboratory Analyses

#### 4.1.1. Routine Laboratory Analyses

A complete blood count (CBC) was performed on an automated hematology analyzer XS-1000i (Sysmex, Kobe, Japan). All biochemical analyses quantifying levels of serum creatinine, aspartate aminotransferase (AST), alanine transaminase (ALT), creatinine kinase (CK), lactate dehydrogenase (LDH), and C-reactive protein (CRP) were performed on a Cobas Integra 400 Plus II analyzer (Roche Diagnostics Corporation, Indianapolis, IN, USA).

#### 4.1.2. ABO Blood Typing

The ABO blood type was determined by performing forward and reverse complete grouping of a K2EDTA-treated venous blood sample via the Gel card technique using DiaClon ABO/D+ Reverse Grouping and an ABD-Confirmation assay (Bio-Rad, DiaMed GmbH, Cressier, Switzerland).

### 4.2. Serological Analyses

#### 4.2.1. Detection and Quantification of Allergen-Specific IgE

The concentration of IgE specific to the following allergens (alpha-GAL (o215), beef (f27), pork (f26), rabbit meat (f213), mutton/lamb (f88), chicken meat (f83), meat turkey (f284), and beef gelatin (c74)) was measured in the patients’ serum.

The serum concentration of allergen-specific IgE was measured by the ImmunoCAP method with the Phadia 200 system (Thermo Fisher Scientific, Waltham, MA, USA). The manufacturer’s procedure was fully followed. The ImmunoCAP is a certified in vitro diagnostic (CE IVD) test system for the quantitative measurement of allergen-specific IgE in human serum or plasma. The ImmunoCAP system uses the FEIA (fluoro-immuno-enzymatic) method. During the analytical procedure, the allergen-specific IgE from the patient’s serum reacts with the corresponding allergens that are covalently bound to ImmunoCAP. After washing non-specific (unbound) particles from the patient’s serum, an enzyme-labeled anti-IgE conjugate (mouse monoclonal anti-human IgE labeled with β-galactosidase) is added to detect specific IgE bound by allergens coated on ImmunoCAP. Excess unbound conjugate (enzyme-labeled anti-IgE) is washed out from the reaction medium, and then a development reagent (4-Methylumbelliferyl-ß-D-galactoside) is added to detect the complexes formed on ImmunoCAP. The enzymatic reaction is stopped by introducing 4% sodium carbonate into the reaction medium. The automatic detection of the chemiluminescent signal is converted by the analyzer software into the value of the specific IgE concentration in the test sample. The automatic detection of the chemiluminescent signal is converted by the analyzer software into the concentration of specific IgE in the tested sample. The concentration is expressed in kUA/L. The method is calibrated in the range of 0–100 kUA/L (reference material: the IgE calibrators are traceable (via an unbroken chain of calibrations) to the 2nd International Reference Preparation (IRP) 75/502 of Human Serum Immunoglobulin E from World Health Organization (WHO)). The overall limit of quantitation for allergen-specific IgE antibodies is 0.1 kUA/l. The cross-reactivity with other human immunoglobulins is non-detectable at physiological concentrations of IgA, IgD, IgM, and IgG.

#### 4.2.2. Quantification of Total IgE

The concentration of total IgE in venous blood serum was determined using the ImmunoCAP Total IgE method using the Phadia 200 system (Thermo Fisher Scientific, Waltham, MA, USA). ImmunoCAP Total IgE is an in vitro assay system (CE IVD) for the quantitative measurement of total IgE circulating in human serum or plasma. The test was performed entirely in accordance with the manufacturer’s procedure. Anti-IgE (mouse monoclonal antibodies), covalently bound to ImmunoCAP, reacts with total IgE in the patient sample. After washing, enzyme-labeled anti-IgE antibodies (mouse anti-human-anti-IgE labeled ß-Galactosidase) are added to form the complex. Following incubation, the unbound anti-IgE enzyme is washed away and the bound complex is then incubated with the developing agent (4-Methylumbelliferyl-ß-D-galactoside). After stopping the reaction (sodium carbonate 4%), the fluorescence of the eluate is measured. The fluorescence is directly proportional to the concentration of IgE in the sample. The higher the response, the more IgE is present in the sample. To evaluate the test results, patient sample responses are converted to concentrations using a calibration curve. The calibration curve range is 2–5000 kU/L (reference material: The IgE calibrators are traceable (via an unbroken chain of calibrations) to the 2nd International Reference Preparation (IRP) 75/502, or the equivalent 3rd International Standard 11/234, of Human Serum Immunoglobulin E from World Health Organization (WHO)). The detection limit is 2 kU/L.

#### 4.2.3. Detection and Quantification of Anti-Borrelia and Anti-Rickettsia IgM and IgG

The serum samples of both patients were inactivated at 56 °C and were used for the detection of seroreactivity to tick-borne pathogens. For anti-*Borrelia* spp. IgG and IgM reactivity, a commercial ELISA kit was used (recomWell *Borrelia* IgG and IgM, Mikrogen Diagnostik GmbH, Neuried, Germany; Cat. No. 4204, 4205). The number of units/mL was calculated according to the manufacturer’s instructions after measuring the optical density (O.D.) at 450 nm. When *Borrelia*-reactive antibodies were detected via ELISA, a second-tier test (recomLine *Borrelia* IgM, IgG, Mikrogen Diagnostik GmbH, Neuried, Germany; Cat. No. 4272, 4273) was used for confirmation, according to manufacturer instructions. For the quantification of anti-*Rickettsia* IgM and IgG, a commercial ELISA kit was used (Vircell S.L., Grenada, Spain, Cat. No. G/M1005), which included well plates coated with cells containing *Rickettsia conorii*. The results were interpreted according to the manufacturer’s instructions after measuring the optical density (O.D.) at 450 nm using an ELX800 ELISA reader (BioTek, Wisconsin, VT, USA).

### 4.3. Molecular Detection of Vector-Borne Pathogens

#### 4.3.1. DNA Extraction

The total DNA was extracted from the skin biopsy sample, the buffy-coat blood, and the complete blood using a Nucleospin Tissue kit (Macherey Nagel, Düren, Germany) according to the manufacturer’s instructions. The purified DNA was eluted into 50 μL elution buffer and stored at −80 °C until further processing.

#### 4.3.2. DNA Pre-Amplification and Microfluidic Real-Time PCR

To improve the detection of pathogen DNA, the total DNA was pre-amplified using a PreAmp Master Mix (Fluidigm, CA, USA) according to the manufacturer’s instructions. Primers targeting all pathogens (see next section) were pooled by combining an equal volume of each primer for a final concentration of 200 nM. The reaction was performed in a final volume of 5 μL containing 1 μL Perfecta Preamp 5X, 1.25 μL pooled primer mix, 1.5 μL distilled water, and 1.25 μL DNA. The thermocycling program consisted of one cycle at 95 °C for 2 min, 14 cycles at 95 °C for 15 s, and 4 min at 60 °C. At the end of the cycling program, the reactions were diluted 1:10 in Milli-Q ultrapure water. All the pre-amplified DNA samples were stored at −20 °C until needed.

To detect major TBPs, 27 bacterial species (*B. burgdorferi* s.s., *B. garinii*, *B. afzelii*, *B. valaisiana*, *B. lusitaniae*, *B. spielmanii*, *B. bissettii*, *B. miyamotoi*, *Anaplasma marginale*, *Anaplasma platys*, Anaplasma phagocytophilum, Anaplasma ovis, Anaplasma centrale, *Anaplasma bovis*, *Ehrlichia canis*, *Neoehrlichia mikurensis*, *R. conorii*, *R. slovaca*, *R. massiliae*, *R. helvetica*, *R. aeschlimannii*, *R. felis*, *Bartonella henselae*, *Francisella tularensis*, *Francisella-like endosymbionts*, *Coxiella-like endosymbionts*, and *Coxiella burnetii*), 7 parasite species (*Babesia microti*, *Babesia canis*, *Babesia ovis*, *Babesia bovis*, *Babesia caballi*, *Babesia venatorum*, and *Babesia divergens*), 5 bacterial genera (*Borrelia*, *Anaplasma*, *Ehrlichia*, *Rickettsia*, and *Mycoplasma*), and 3 parasite taxa (*Apicomplexa*, *Theileria*, and *Hepatozoon*), the BioMark™ real-time PCR system (Fluidigm, CA, USA) was used for high-throughput microfluidic real-time PCR amplification using 48.48 Dynamic Array™ IFC chips (Fluidigm, CA, USA). These chips dispense 48 PCR mixes and 48 samples into individual wells, after which on-chip microfluidics assemble real-time PCR reactions in individual chambers before thermal cycling, resulting in 2304 individual reactions. Briefly, amplifications were performed using 6-carboxyfluorescein (FAM)- and black hole quencher (BHQ1)-labeled TaqMan probes with the TaqMan Gene expression master mix according to the manufacturer’s instructions (Applied Biosystems, Courtaboeuf, France). PCR cycling included 2 min at 50 °C and 10 min at 95 °C, followed by 40 cycles of two-step amplification of 15 s at 95 °C, and 1 min at 60 °C. One negative water control was included per chip. To determine whether factors present in the sample could inhibit the PCR, Escherichia coli strain EDL933 DNA was added to each sample as an internal inhibition control, and primers and a probe specifically for the Escherichia coli intimin gene (eae) were used. For more details regarding the development of this new high-throughput tool based on real-time microfluidic PCRs (test of sensitivity, specificity, and controls used), please see [31,32].

### 4.4. Molecular Analysis Genetic of Markers Associated with Allergic Diseases

#### 4.4.1. HLA Class I and II Genotyping

HLA class I and II alleles were genotyped using sequence-specific oligonucleotides (PCR-SSOs). Diluted DNA samples were amplified via PCR using the GeneAmp^®^ PCR System 9700 (Applied Biosystems, Waltham, MA, USA). The PCR products were then hybridized against a panel of oligonucleotide probes on coated polystyrene microspheres. These microspheres had sequences complementary to polymorphic stretches within the target HLA class I and II alleles, using the Lifecodes HLA SSO typing kits (Immucor, West Avenue, Stamford, CT, USA). Detection was performed using the Luminex^®^ IS 100 system (Luminex Corporation, Austin, TX, USA). The results of HLA class I and II allele genotyping were evaluated using the MATCH IT DNA software v1.3 (One Lambda, Canoga Park, CA, USA).

### 4.5. Histological Analysis of Skin Lesion

A skin biopsy was collected from the lesion and fixed in 10% formalin. The fixed tissue was then embedded in paraffin. Thin sections of the paraffin-embedded tissue were prepared and subjected to an automated processing procedure using the Epredia™ Excelsior™ AS Tissue Processor (Thermo Fisher Scientific, Inc.) following the manufacturer’s recommended protocol. The tissue sections were subsequently stained with hematoxylin and eosin (H&E) solution, a process that lasted for 90 min at 21 °C.

## Figures and Tables

**Table 1:** Titers of allergen-specific and total IgE in examined patients.

Allergen	Allergen Code	Patient I (Skopje)	Patient II (Novi Sad)
		Specific IgE *	
α-Gal	o215	<0.1 kUA/L	0.20 kUA/L
Pork	f26	<0.1 kUA/L	<0.1 kUA/L
Beef	f27	<0.1 kUA/L	<0.1 kUA/L
Mutton	f88	<0.1 kUA/L	<0.1 kUA/L
Beef gelatin	c74	<0.1 kUA/L	<0.1 kUA/L
Rabbit (meat)	f213	<0.1 kUA/L	<0.1 kUA/L
Chicken (meat)	f83	<0.1 kUA/L	<0.1 kUA/L
Turkey (meat)	f284	<0.1 kUA/L	<0.1 kUA/L
Total IgE **		3.99 kU/L	10.00 kU/L
α-Gal (sIgE)/total IgE ratio (%)			2%

* Specific IgE reference values; 0–0.35 kUA/L (class 0), 0.35–0.70 kUA/L (class 1), 0.7–3.50 kUA/L (class 2), 3.5–17.50 kUA/L (class 3), 17.5–50.00 kUA/L (class 4), 50.00–100.00 kUA/L (class 5), >100 kUA/L (class 6). ** Total IgE reference values; Adults: <115 IU/mL, Children: 8–9 years <78 IU/mL; 9–10 years <85 IU/mL; >10 years. <115 IU/mL.

**Table 2:** HLA class I and II gene distribution in investigated patients.

Patient	HLA Class I			HLA Class II	
	HLA A	HLA B	HLA C	HLA DRB1	HLA DQB1
Patient I (Skopje)	*11,*30	*13,*44	*05,*06	*07,*13	*02,*06
Patient II (Novi Sad)	*02,*66	*27,*52	*02,*12	*01,*15	*05,-

Legend: * correspond to the antigen specificity and the assigned HLA number.

## Data Availability

All data generated in this manuscript are available in the main text.
